# Computational delineation and quantitative heterogeneity analysis of lung tumor on 18F-FDG PET for radiation dose-escalation

**DOI:** 10.1038/s41598-018-28818-8

**Published:** 2018-07-13

**Authors:** Xiuying Wang, Hui Cui, Guanzhong Gong, Zheng Fu, Jianlong Zhou, Jiabing Gu, Yong Yin, Dagan Feng

**Affiliations:** 1 BMIT research group, School of Information Technologies, The University of Sydney, Sydney, Australia; 2 The Radiation Oncology Department of Shandong Cancer Hospital, Affiliated to Shandong University, Jinan, China; 3 PET/CT center, Shandong Tumor Hospital and Institute, Shandong Academy of Medical Sciences, Jinan, China; 4 Data 61, CISRO, Sydney, Australia; 5 Med-X Research Institute, Shanghai Jiao Tong University, Shanghai, China

## Abstract

Quantitative measurement and analysis of tumor metabolic activities could provide a more optimal solution to personalized accurate dose painting. We collected PET images of 58 lung cancer patients, in which the tumor exhibits heterogeneous FDG uptake. We design an automated delineation and quantitative heterogeneity measurement of the lung tumor for dose-escalation. For tumor delineation, our algorithm firstly separates the tumor from its adjacent high-uptake tissues using 3D projection masks; then the tumor boundary is delineated with our stopping criterion of joint gradient and intensity affinities. For dose-escalation, tumor sub-volumes with low, moderate and high metabolic activities are extracted and measured. Based on our quantitative heterogeneity measurement, a sub-volume oriented dose-escalation plan is implemented in intensity modulated radiation therapy (IMRT) planning system. With respect to manual tumor delineations by two radiation oncologists, the paired t-test demonstrated our model outperformed the other computational methods in comparison (p < 0.05) and reduced the variability between inter-observers. Compared to standard uniform dose prescription, the dosimetry results demonstrated that the dose-escalation plan statistically boosted the dose delivered to high metabolic tumor sub-volumes (p < 0.05). Meanwhile, the doses received by organs-at-risk (OAR) including the heart, ipsilateral lung and contralateral lung were not statistically different (p > 0.05).

## Introduction

Lung cancer is a major cause of cancer death worldwide^1^. The two major types of lung cancer are non-small cell lung cancer (NSCLC) and small cell lung cancer (SCLC). For SCLC patients, chemotherapy is an essential treatment procedure and radiation therapy (RT) may be a treatment option depending on the stage of cancer^2^. Treatment for patients with NSCLC may include various combinations of surgery, RT, chemotherapy and immunotherapy^2–4^, Most early-stage NSCLC patients are treated with surgery or stereotactic body radiotherapy (SBRT) that often has similar outcomes to surgery^4^. Chemotherapy in combination with radiation treatments are typically recommended for patients with stage III lung cancer^2^. In stage IV NSCLC, the choice of treatment depends on many factors and patients may also benefit from target radiation to residual lesions^2^. Thus, RT plays an essential role in lung cancer treatment. Nevertheless, there could be local tumor recurrence with RT. The mechanisms are unclear but they may relate to tumor heterogeneity, i.e differences in levels of hypoxia, cell density, tumor genetics, proliferation and vascularization^2,3,5–8^. Therefore, identification and analysis of tumor heterogeneity would be beneficial to tailoring treatment with RT in patients with lung cancer^9,10^.

Positron emission tomography (PET) is a functional imaging modality and plays an essential role in the diagnosis and staging of lung cancer. The primary clinical outcomes obtained from PET guided RT include target volume definition, nodal disease determination, RT planning modification, treatment response prediction and prognosis judgment^10–12^. For Fludeoxyglucose ([^18^F]FDG)-PET, the volumes of local relapse are correlated with high uptake regions which are treatment resistant areas^13^. In RT, to deal with the local recurrence, one solution is to deliver increased dose to high [^18^F]FDG uptake regions while maintaining the dose to other parts of the heterogeneous tumor by dose painting. Intensive research has investigated the relations between tumor sub-volumes with different metabolic activities and the survival outcome^12,14–16^. It has been proved that the localization and analysis of sub-volumes in metabolic tumor volume (MTV) are necessary for dose painting and precision personalized RT treatment planning^9^ using [^18^F]FDG- and [^18^F]flortanidazole (HX4)- PET/Computed Tomography(CT) imaging^12–16^.

The usual approach to define the boundaries of MTV and sub-volumes is by manual delineation. Manual delineation, however, is time consuming, labour intensive, operator-dependent^17^ and subject to inter- and intra-observer variations^18,19^. Most of the existing computational segmentation methods^17,20^ including our previous work^21,22^ focused on the overall tumor segmentation for accurate cancer diagnosis purposes. As the delineation of complete tumor volume together with the measurement and extraction of metabolic sub-volumes from [^18^F]FDG-PET images are the basis of effective personalized treatment planning, and in order to better interpret the different levels of requests from diagnosis and treatment aspects, we propose an automated computational technique that delineates the target tumor and in the meanwhile, identifies and measures metabolic sub-volumes. A dose-escalation plan using the computational delineation and measurement results is performed by intensity modulated radiation therapy (IMRT) system. The technique is proposed with hope to provide second opinion for personalized precision RT treatment planning with radiation dose-escalation based on [^18^F]FDG-PET.

## Materials and Methods

### Patient studies

58 [^18^F]FDG-PET/CT scans were retrospectively acquired in lung cancer patients. All patients were scanned on a Discovery LS PET-CT system (GE Healthcare, Milwaukee, WI, USA) after the injection of [^18^F]FDG. The patient studies enrollment criteria include: patients who received 60Gy (standard dose) and concurrent chemotherapy but had local recurrence; tumor exhibited inhomogeneous intensity distributions or indistinct boundaries. Among the patient studies, there are 30 cases with tumor infiltrating chest wall, 21 cases with tumor adjacent to mediastinum and 7 isolated cases. The PET images were reconstructed using a matrix of 144 × 144 pixels with a size of 4 mm × 4 mm × 4 mm; the CT images were reconstructed using a matrix of 512 × 512 pixels with a size of 1.17 mm × 1.17 mm × 5 mm. The PET images were up-sampled to the size of CT to obtain a spatial correspondence between the volumes in a common space. Manual tumor delineations were drawn on the registered PET with CT images as reference by two radiation oncologists (referred to as Obs-1 and Obs-2) for automated segmentation accuracy evaluation.

All the data used in this study were approved by Medical Ethics Approval Committee at Shandong Cancer Hospital. All the patients consented to participate in this study and have signed a written informed consent document. The methods were performed in accordance with relevant guidelines and regulations.

### Automated target tumor segmentation

**Figure 1:**
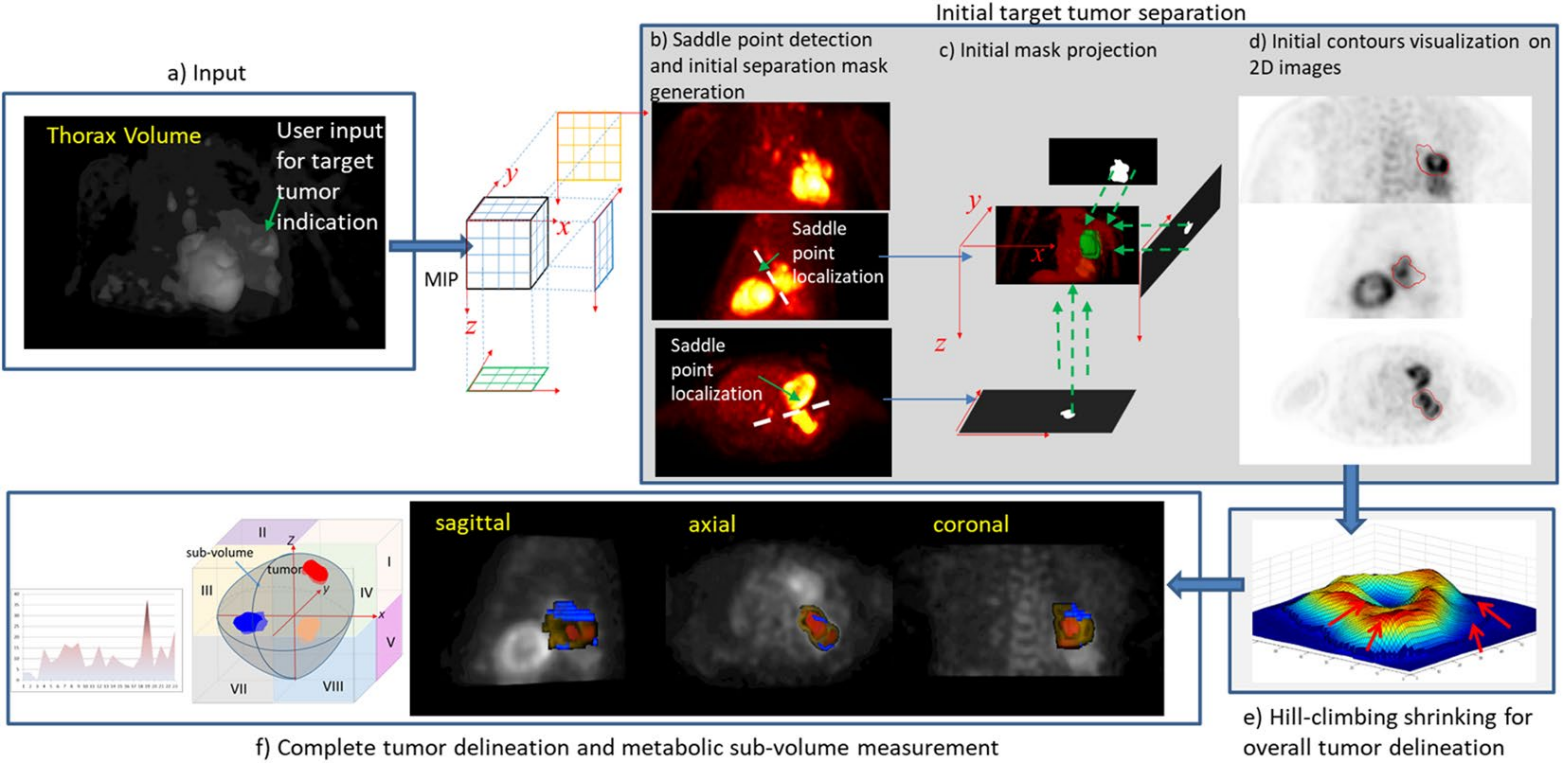
Outline of the proposed method. The max intensity projection (MIP) images of (**a**) input thorax volume are firstly extracted. (**c**) The separation masks are obtained by partitioning (**b**) the MIP images. (**d**) 3D-MS of the reconstructed ROI (green object in (**c**)) is shown on coronal, sagittal and axial views. (**e**) The overall tumor segmentation by a hill-climbing shrinking method starting from 3D-MS. (**f**) 3D visualization of the segmented tumor volume; red, yellow and blue colour maps indicate high, moderate and less active sub-volumes.

The proposed tumor segmentation framework is given in Fig. 1. The first step is to separate the target tumor from surrounding structures by extracting a 3D masking surface (3D-MS). Secondly, a tumor customized boundary delineation method is proposed where the 3D-MS will shrink in a hill-climbing manner towards the tumor center until a joint stopping criterion is satisfied.

#### Stage 1

The first stage is to initially extract a 3D-MS for tumor separation. As high FDG uptake tissues such as tumor appear as salient regions standing out from the background, given an input thorax volume and user defined seed for target tumor localization, max intensity projection (MIP) images are firstly generated along x, y and z coordinate directions. For the cases (as shown in Fig. 1(b)) where the tumor is attached to neighboring high uptake regions such as the heart, a saddle point where two salient regions attached is firstly located on each MIP image. Then the corresponding tangent line is generated to separate neighboring attached salient regions. The isocontour (Fig. 1(d)) passing through the saddle point is used to obtain a region of interest (ROI) mask. Finally, the seeds and ROI masks obtained from each of the MIP images are projected back to 3D space to generate 3D-MS.

#### Stage 2

Given the detected 3D-MS, the second stage is to shrink the surface towards the true tumor boundary. The aim of tumor segmentation can be considered as to assign each of the voxels inside 3D-MS a foreground/tumor label *l*_*F*_ or background label *l*_*B*_. To obtain the shrinking stopping criterion, we define the joint GM and intensity affinity of two adjacent voxels v_*i*_, v_*j*_ as

1 $$\varphi ({v}_{i},{v}_{j})=exp(-\beta (g({v}_{j})-g({v}_{i})))$$

where the intensity-based affinity term $\beta  > 0$ when $\overline{{f}_{i}} > \,\overline{{f}_{j}}$ where $\overline{{f}_{i}}$ denotes the average SUV value *f* within a neighboring box centered at v_*i*_; *β* < 0 otherwise. *g* = *G*⋅∇*f* is Gaussian filtered GM where ∇*f* denotes the GM value, $G=\frac{exp(\frac{1}{2{\sigma }^{2}}{\sum }_{i\mathrm{=1}}^{3}{d}_{i}^{2})}{{\mathrm{(2}\pi )}^{\frac{3}{2}}{\sigma }^{3}}$ is a rotationally symmetric Gaussian low pass-filter where *σ* is the standard deviation, *d*_*i*_ denotes the distance from the origin in axis. The filter is applied to GM image to reduce the effect of noise while preserving the salient boundaries. In our experiments, *β* is set as 60 (−60) when $\beta  > 0$ (*β* < 0) and *σ* is set as 1.5. It is noted that the absolute value of *β* in Equation (1) has no effect on the segmentation results because what matters is the condition of *β* being positive or negative. With *ϕ* defined as Equation (1), the segmentation is implemented in an iterative searching process by Algorithm 1.

**Algorithm 1:**
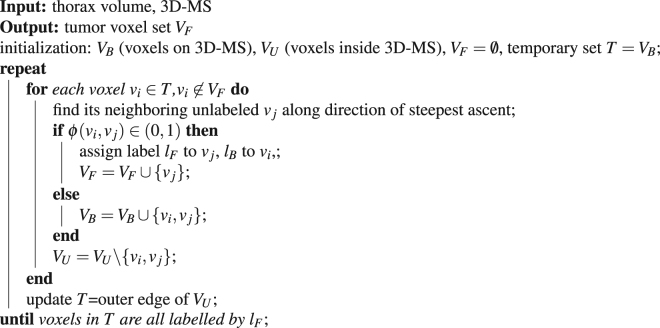
Pseudocode of the hill-climbing shrinking algorithm.

#### Segmentation accuracy evaluation

To evaluate the segmentation accuracy of the proposed method, we calculate the spatial overlap and shape dissimilarity between the automated segmented tumor volume (TV_*auto*_) and manually segmented tumor volume (TV_*manual*_) by Dice similarity coefficient(DSC) and Hausdorff distance (HD) respectively. DSC and HD are defined as:

2 $$DSC(T{V}_{auto},T{V}_{manual})=\frac{\mathrm{2|}T{V}_{auto}\cap T{V}_{manual}|}{|T{V}_{auto}|+|T{V}_{manual}|}$$

3 $$HD(T{V}_{auto},T{V}_{manual})=max\{\mathop{{\rm{\sup }}}\limits_{i\in {S}_{auto}}\mathop{{\rm{\inf }}}\limits_{j\in {S}_{manual}}d(i,j),\mathop{{\rm{\sup }}}\limits_{j\in {S}_{manual}}\mathop{{\rm{\inf }}}\limits_{i\in {S}_{auto}}d(i,j)\}$$

where *S* denotes the surface of a volume, $\sup$ represents the least subset element and $\inf$ denotes the greatest subset element; *d* is the Euclidean distance between points *i* and *j*. The DSC value is 1 for a perfect segmentation and a low HD value indicates high segmentation accuracy.

The performance of our method was compared with (1) a threshold of 40 % SUV_*max*_, referred to as RG40; (2) a threshold of 50 % SUV_*max*_, referred to as RG50; (3) iterative fuzzy c-means (FCM) clustering^23^, (4) a tumor-customized downhill model for PET segmentation (TCD)^24^, (5) a distance regularized level set evolution (LSE)^25^, (6) an active contour for lung tumor segmentation (LAC)^26^; (7) random walk (RW)^27^ and (8) a local adaptive RW for PET segmentation (LARW)^28^. All the methods are implemented with MATLAB R2017a on a PC with 3.50 GHZ Intel (R) Core (TM) i7-4770K CPU and 16.GB memory, running a 64-bit Windows operating system. RG40, RG50, FCM and TCD were implemented within a fixed bounding box of 60 × 60 × 20 voxels centred at the initial seed. The user seed was provided by a person with knowledge to identify a tumor.

### Quantitative heterogeneity computing for dose-escalation

#### Heterogeneous sub-volume extraction and measurement

Metabolic sub-volumes are further segmented within our segmented tumor volume (TV_*PM*_) for heterogeneity measurement and dose-escalation purposes. We partition tumor sub-volumes with low, moderate and high metabolic activities (referred to as TV_*low*_, TV_*mod*_ and TV_*high*_) using respective SUV ranges^29^ of (0,25%]SUV_*max*_, (25%,50%]SUV_*max*_ and (50%,100%]SUV_*max*_. According to the calculation grid voxel size of the mainly used Treatment Planning System (TPS)^30^, a sub-volume whose size is smaller than 5 × 5 × 1 voxels is merged to the range of its nearest sub-volume and not counted as one isolated sub-volume.

#### Dose-escalation based on FDG-PET driven dose painting

The segmented tumor and metabolic sub-volumes are tested on Eclipse (Varian Medical Systems, version13.5) treatment planning system for dose-escalation in FDG-PET driven dose painting. Two six-field inverse-planned IMRT treatment plans are designed. One plan (plan A) is with uniform dose prescription of the entire tumor volume, the other comparative plan (plan B) is with sub-volume oriented dose-escalation based on our extracted metabolic sub-volumes. These two plans are set as:

- Plan A: a 10-mm margin is added to GTV_*manual*_ to create the planning target volume (PTV_*planA*_). PTV_*planA*_ is prescribed a dose ${D}_{PT{V}_{planA}}$ where $60Gy < {D}_{{PTV}_{planA}} < 66Gy$;
- Plan B: PTV_*planB*_ is obtained as PTV_*planB*_ = PTV_*high*_ + PTV _*mod*_ + PTV_*low*_ where PTV_*high*_ (PTV_*mod*_, PTV_*low*_) is generated by applying a 10-mm margin to TV_*high*_ (TV_*mod*_, PTV_*low*_). The prescribed dose settings are: $60Gy < {D}_{{PTV}_{low}} < 70Gy,\,70Gy < {D}_{{PTV}_{mod}} < 80Gy,{D}_{{PTV}_{high}} > 80Gy$.

#### Treatment plan evaluation

To compare the two treatment plas A and B, we calculate the dose distribution and dose-volume histogram (DVH) of PTV and organ-at-risk (OAR). OAR is manually delineated by experts on the planning PET/CT which includes the heart, ipsilateral lung, contralateral lung and spine cord.

Two heterogeneity indexes (HI), HI_*RTOG*_ = *I*_*max*_/*RI* and HI_*D*2,*D*98_^31^ = (*D*_2%_ − *D*_98%_)/*D*_*p*_ × 100, are calculated to evaluate the two plans, where *I*_*max*_ = maximum isodose in the target, RI = reference isodose, *D*_2%_ and *D*_98%_ represent the near-maximal and near-minimal dose respectively. If HI_*RTOG*_ ≤ 2, the treatment plan is considered to comply with the protocol. A lower HI_*D*2,*D*98_ value indicates a less heterogeneous target dose.

## Results

### Automated tumor segmentation performance

The inter-observer variation between obs-1 and obs-2 was 0.824 ± 0.079 with respect to DSC and 8.99 ± 6.67 mm with respect to HD. As shown by Table 1, the proposed method (PM) achieved the best tumor segmentation results with DSCs of 0.850 ± 0.044 and 0.847 ± 0.052, HDs of 7.47 ± 3.14(mm) and 7.38 ± 2.71(mm), followed by LSE and LARW. The improvement was statistically significant (*p* < 0.05). The computation time of the whole procedure by PM depends on the size of the lesion and was about 0.82 seconds for one slice. The time taken in initial tumor separation was about 0.68 second per case. The average computation time in tumor segmentation was 0.63 for one slice.

**Table 1:** Segmentation evaluation results by DSC and HD (mm).

Method	Obs-1				Obs-2			
	DSC		HD(mm)		DSC		HD(mm)	
	mean (SD)	*p*-value	mean (SD)	*p*-value	mean (SD)	*p*-value	mean (SD)	*p*-value
RG40	0.68 (0.18)	2.2E-07	25.12 (16.56)	3.1E-05	0.71 (0.26)	4.2E-07	26.51 (18.06)	6.0E-06
RG50	0.58 (0.21)	5.3E-05	22.35 (14.13)	1.8E-06	0.62 (0.20)	6.5E-05	22.11 (14.01)	4.3E-05
FCM	0.69 (0.17)	4.7E-05	34.93 (18.76)	4.8E-06	0.73 (0.19)	5.5E-06	34.05 (21.00)	2.6E-07
TCD	0.63 (0.20)	6.1E-04	19.69 (15.20)	8.4E-05	0.67 (0.25)	5.2E-06	19.12 (15.18)	9.7E-04
LSE	0.80 (0.21)	1.1E-06	17.85 (8.34)	9.5E-05	0.81 (0.27)	3.7E-06	15.28 (9.06)	6.8E-05
RW	0.76 (0.52)	1.9E-05	18.61 (13.06)	1.1E-04	0.78 (0.23)	8.5E-04	17.51 (10.02)	2.5E-04
LARW	0.80 (0.17)	9.6E-04	13.19 (5.73)	8.1E-03	0.81 (0.25)	8.6E-04	12.87 (9.63)	9.9E-03
PM	**0.84** (**0.04)**	—	**8.61** (**3.12)**	—	**0.85** (**0.09)**	—	**10.47** (**6.54)**	—

**Figure 2:**
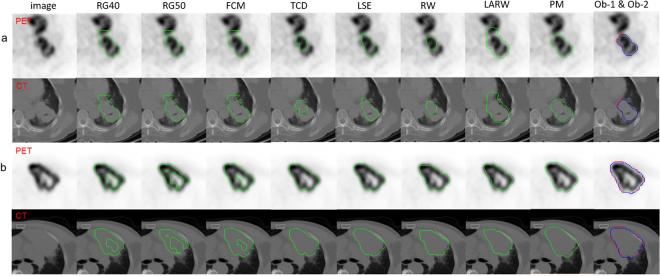
Two segmentation results shown in cropped PET (inverted for display) and CT images. The window width (W) and level (L) of the two sets of CT images are W: 350, L:35.

Two cases are shown in Fig. 2 where the tumor in Fig. 2(a) is with irregular margins and lies adjacent to the myocardium with similar FDG uptake. The local tumor extent is difficult to discern on PET. RG40, RG50, FCM and LARW failed in the tumor separation while LSE resulted in smaller tumor delineation and RW failed to delineate the whole tumor. PM was able to accurately separate the complete tumor from the surrounding structures with the DSC of 0.858 (Obs-1) and 0.831 (Obs-2), HD of 9.179 mm (Obs-1) and 9.356 mm (Obs-2) respectively. For the case in Fig. 2(b), the tumor has heterogeneous FDG uptake reflecting regions of necrosis. RG40, RG50, FCM excluded the tumor regions with low SUVs and failed to delineate the whole tumor. PM obtained the best tumor delineation with DSC of 0.943 (Obs-1) and 0.935 (Obs-2), as well as HD of 8.940 mm (Obs-1) and 6.939 mm (Obs-2) respectively. TCD and LARW resulted in smaller tumor segmentation.

### Heterogeneity measurement and dose-escalation analysis

**Figure 3:**
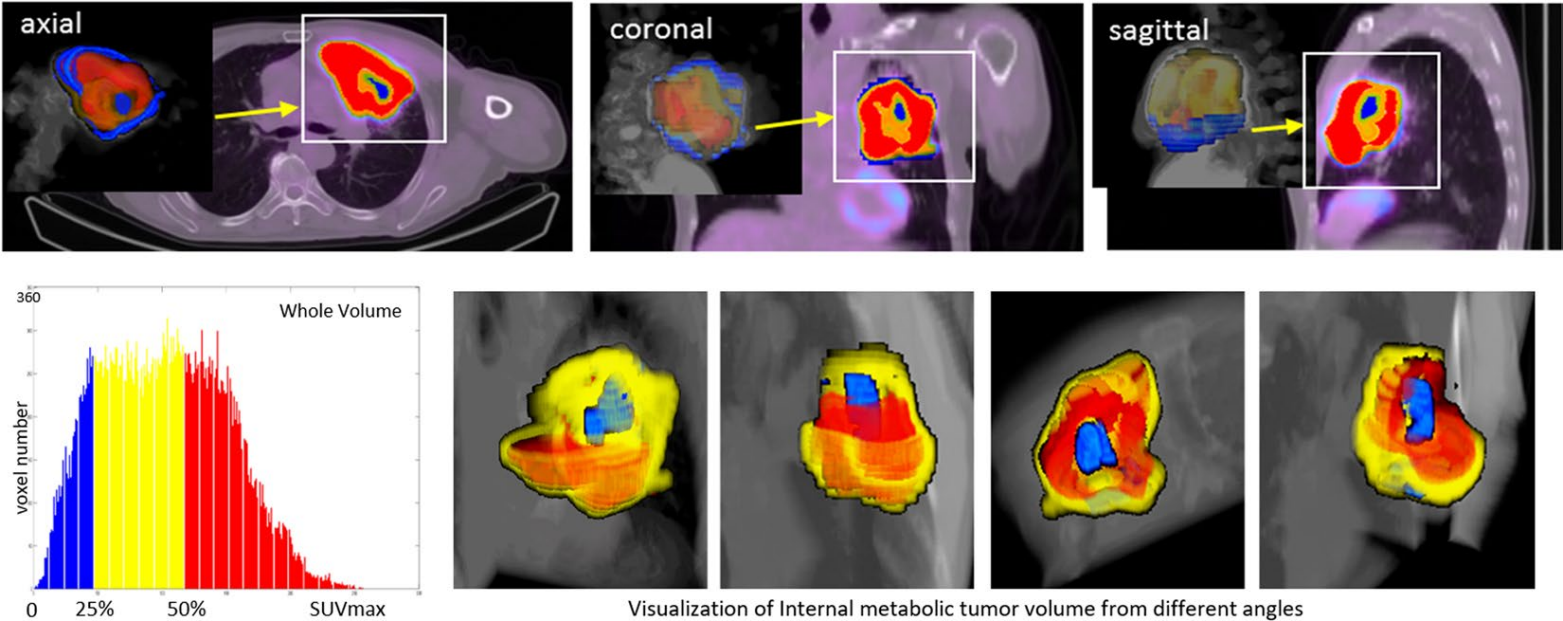
The extraction and measurement of metabolic sub-volumes and spatial SUV distributions of the case in Fig. 2(b). PTV_*high*_, PTV_*mod*_ and PTV_*low*_ are shown by red, yellow and blue colors respectively.

The case in Fig. 2(b) with high heterogeneity is visually and quantitatively measured and analyzed as shown by Fig. 3. In this squamous cell carcinoma case, red, yellow and blue color maps are used to indicate the location and spatial relations of TV_*high*_, TV_*mod*_, TV_*low*_ in the ranges of (50%, 100%], (25%, 50%] and %(0, 25%] SUV_*max*_. The TV_*low*_ s located near the tumor boundary were small, however, the intensities were greater than 23.86 % SUV_*max*_ which could be considered as an acceptable value for tumor definition in clinical practice^32^. In contrast, the SUV values of the other three larger TV_*low*_ s located closer to the tumor centre were as low as 8.24 % SUV_*max*_. It can be learnt from the spatial SUV distributions that there were significant variations between the distributions of high, moderate and less active sub-volumes within this tumor.

**Figure 4:**
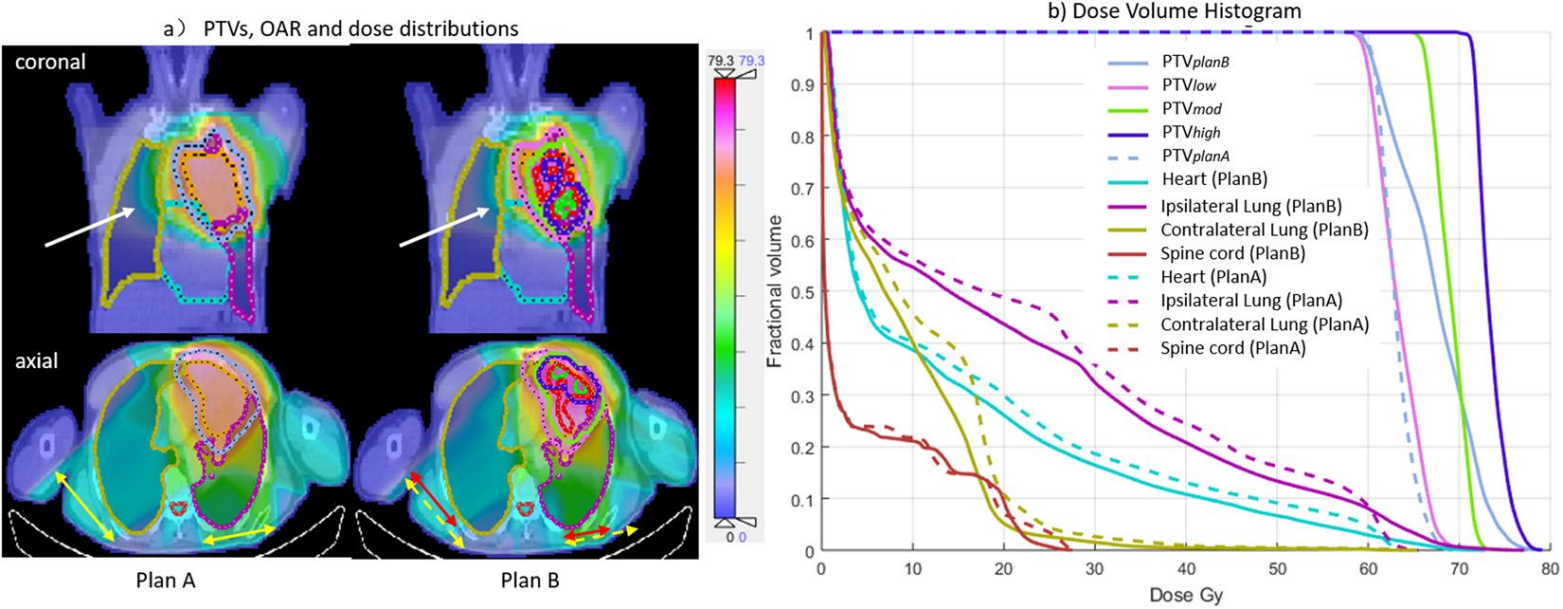
Dose-Volume histogram (DVH) for the Planning target volume (PTV) and the organ at risk volume (OAR) in treatment plans A and B of the case in Fig. 3. Compared with plan A, the isodose contour of 20Gy (as pointed by the white arrow in coronal view) is much closer to the target volume and the radiation field width becomes shorter (as compared by red and yellow arrows) in plan B.

For the case analyzed in Fig. 3, its DVH and isodose distributions of treatment plans A and B are given in Fig. 4. The tumor was located in the left upper lung and had denser and higher dose distributions in plan B than comparative plan A. As pointed by the white arrows in Fig. 4(a), the isodose contour of 20 Gy in plan B is much closer to the target volume than plan A, which indicated a lower dose received by contralateral lung. A decrement of radiation field width can also be observed in axial view. As shown by DVH in Fig. 4(b), plan B decreased the volume fractions of OAR when the isodose value was between 10 Gy and 60 Gy.

The dosimetry results of seven patients (characteristics listed in Table 2) with respect to PTV and OAR are given in Tables 3 and 4. All these cases complied with the protocol (HI_*RTOG*_ ≤ 2). The PTV homogeneity was higher in plan A. As shown by Table 3, plan B resulted in a significant boost to the maximal (D_2_) and average (D_*mean*_) doses received by PTV (*p*-values < 0.05). The doses received by OAR in plan B (shown by Table 4) were not statistically significantly different from that in plan A (*p*-values > 0.05).

**Table 2:** Patient and tumor characteristics of seven male patients.

ID	Location	Age	TNM	Stage	Pathology	PTV	PTV_*low*_		PTV_*mod*_		PTV_*high*_	
						ml	ml	%	ml	%	ml	%
1	L-U	68	T4N2M0	3b	Squamous cell carcinoma	598.47	255.70	42.73	230.20	38.46	112.57	18.81
2	L-U	68	T4N1M0	3a	Small cell carcinoma	618.05	243.16	39.34	201.39	32.59	173.50	28.07
3	L-L	60	T2N1M0	2a	Adenocarcinoma	361.53	126.03	34.86	147.22	40.72	88.28	24.42
4	R-L	40	T2N2M1	4	Small cell carcinoma	968.54	563.77	58.21	442.76	45.71	37.99	3.92
5	M	59	T4N3M1	4	Adenocarcinoma	457.75	148.72	32.49	177.70	38.82	131.33	28.69
6	L-M	73	T4N3M0	3b	Squamous cell carcinoma	394.16	228.06	57.86	112.64	28.58	53.46	13.56
7	R-U	63	T4N0M1	4	Non-small cell carcinoma	874.02	295.91	33.86	506.10	57.90	72.01	8.23

L(R) - U(L) is used to indicate that the tumor is located in the left(right) upper(lower) lobe. M indicates that the tumor is located in the mediastinum, L-M represents the tumor locating in the left lung while being adjacent to the mediastinum.

**Table 3:** Comparison between various metrics in plans A and B with respect to PTV.

ID	D_2_(Gy)		D_98_(Gy)		D_*mean*_(Gy)		HI_*RTOG*_		${\bf{H}}{{\bf{I}}}_{{D}_{2},{D}_{98}}$		V_60_(%)	
	A	B	A	B	A	B	A	B	A	B	A	B
1	67.50	75.55	60.00	59.41	63.13	67.35	1.16	0.99	12.5	20.18	98.02	96.2
2	65.27	89.85	61.01	58.04	63.13	73.39	1.1	1.23	7.1	39.76	99.31	96.17
3	65.49	100.61	58.99	67.31	63.12	83.74	1.11	1.32	10.83	41.63	96.07	99.51
4	65.61	79.61	60.22	62.53	63.11	74.27	1.11	1.05	8.99	21.35	98.35	99.25
5	66.79	84.12	56.39	38.79	62.99	69.08	1.13	1.11	17.33	56.68	93.13	85.96
6	66.66	85.98	58.73	60.47	62.99	75.02	1.12	1.09	9.89	31.89	97.02	98.36
7	64.89	84.69	61.33	67.07	63.13	74.75	1.10	1.10	5.94	22.03	99.89	99.98
*p*-value	—	0.0008	—	0.90	—	0.0015	—	—	—	—	—	—

D_*mean*_ is mean dose; V_60_(%) = PTV volume receiving at least 60Gy; the *p*-value is calculated using two-tailed paired t-test.

**Table 4:** Comparison between various metrics in plans A and B with respect to OAR.

ID	OAR	D_2_(Gy)		D_98_(Gy)		D_*Mean*_(Gy)		V_5_(%)		V_10_(%)		V_20_(%)		V_30_(%)	
		A	B	A	B	A	B	A	B	A	B	A	B	A	B
1	Heart	61.89	62.83	0.93	0.85	15.15	13.74	47.92	46.53	40.11	38.32	30.00	26.26	18.67	16.23
2		45.61	43.55	0.51	0.52	6.66	6.38	27.07	23.58	19.18	16.36	11.23	9.91	5.38	6.89
3		55.75	58.36	6.84	6.72	18.95	21.93	98.89	98.85	77.97	93.35	37.90	50.70	13.90	17.68
4		63.71	70.81	2.26	2.20	20.85	23.12	84.41	82.60	66.12	66.11	36.23	47.52	22.32	26.63
5		62.86	72.79	0.70	0.73	18.69	19.01	57.96	57.65	49.35	48.54	39.12	37.05	27.82	27.39
6		56.06	60.02	0.13	0.16	5.46	5.91	16.69	17.61	13.32	13.68	9.88	10.46	6.34	6.48
7		29.74	32.79	0.38	0.51	4.14	4.67	17.51	19.06	11.81	13.23	4.74	5.53	1.95	2.53
	*p*-value	—	0.05	—	0.74	—	0.26	—	0.35	—	0.50	—	0.34	—	0.28
1	Ipsilateral lung	62.44	66.80	0.90	0.85	22.93	21.27	63.00	61.33	56.55	54.62	48.86	43.79	35.95	32.56
2		63.09	71.53	0.86	0.89	21.61	18.65	50.43	47.32	43.41	39.45	39.06	33.65	35.35	28.06
3		64.17	88.12	0.95	1.04	23.10	27.27	68.84	67.54	60.56	59.44	49.39	48.25	31.81	38.50
4		63.73	77.40	2.75	3.13	38.89	45.57	86.55	89.98	76.03	79.89	69.86	73.02	64.84	68.34
5		63.31	52.63	1.12	0.81	19.63	17.98	64.92	61.54	51.59	47.64	36.65	33.51	26.13	21.49
6		63.46	76.64	0.29	0.33	19.37	22.62	49.06	52.47	42.33	45.85	37.27	40.84	31.74	36.28
7		63.67	78.7	1.09	1.29	21.63	25.49	54.54	57.11	47.07	48.07	40.86	42.38	34.50	36.60
	*p*-value	—	0.06	—	0.54	—	0.28	—	0.99	—	0.77	—	0.54	—	0.92
1	Contralateral lung	33.11	27.75	0.53	0.50	10.54	8.81	60.18	56.85	46.00	40.48	10.94	5.29	2.64	1.53
2		21.38	26.76	0.36	0.40	4.67	5.23	34.14	31.34	13.02	17.66	2.54	4.84	0.31	0.95
3		19.29	19.85	0.72	0.78	5.20	5.84	49.44	52.10	10.50	15.14	1.52	1.94	0.00	0.01
4		22.70	23.92	0.93	0.97	5.05	4.82	24.11	22.22	18.06	15.46	4.01	4.93	0.21	0.64
5		48.90	52.63	0.73	0.81	10.86	11.43	42.81	44.55	32.02	31.91	23.12	23.37	11.42	13.52
6		6.58	4.66	0.06	0.06	0.74	0.69	2.66	1.88	0.78	0.69	0.36	0.30	0.10	0.02
7		42.27	49.27	0.65	0.76	8.43	9.66	35.41	38.77	27	29.11	17.84	20	5.08	6.50
	*p*-value	—	0.39	—	0.05	—	0.71	—	0.89	—	0.77	—	0.96	—	0.27

## Discussion

It has been proved that distinguishing tumor tissues with metabolic heterogeneity would be helpful for tumor customized radiation dose painting and treatment^13,33^. With the development of IMRT, the dose escalation approach called simultaneous integrated boost (SIB) is able to deliver tailored doses to specific tumor sub-volumes with different metabolic levels^29^. Thus the accurate target tumor delineation and metabolic sub-volume extraction are important and essential in dose painting by contours or numbers in order to achieve high-quality RT results^34^.

We proposed an efficient and objective computational technique to outline the complete target tumor volume with heterogeneity, localize and measure tumor sub-volumes of different uptake ranges. The proposed method reduced the variability between inter-observers. Our major findings were: (1) The automatically extracted masking surface from MIP images was able to separate the tumor from its surrounding high FDG uptake regions and also provided an improved initialization for tumor delineation. Using the MIP images, the shape and size of the masking surface and the tumor were coherent. The first stage ensured that a contour is returned which topologically contains the ROI so as to avoid the exclusion of inhomogeneous areas within the tumor. The initial surface incorporated the whole tumor volume and avoided the variability in initialization. For those even more challenging cases when the tumor region has overlapping with surrounding high update regions on all the three projection views of MIP images, the first step with three directions might have difficulty in separation. In this case, we would suggest to increase the number of projection directions in our separation mechanism. (2) The proposed hill-climbing stopping criterion achieved tumor-orientated boundary delineations which complied with manual definitions. This was because the shrinking of the initial masking surface towards the tumor avoided the over-segmentation caused by tumor heterogeneity. In addition, the joint affinity model of GM and intensity was able to obtain a balancing tumor-specified boundary identification. In our experiments over 58 cases, we found that the SUV values of our defined tumor boundaries were between 23.65 % and 37.76 % SUV_*max*_, which also complied with the findings that a value ranging from 15 % to 50 % SUV_*max*_ could be used for tumor delineation^32^.

The quantitative extraction and measurement of tumor heterogeneity provided insightful information of tumor metabolism which assisted inhomogeneous dose calculation and treatment response monitoring and assessment. Our heterogeneity quantitative measurement results were tested on IMRT planning system to perform a dosimetry comparison between a dose-escalation plan and a standard uniform dose prescription plan. The evaluation results of seven patient studies demonstrated that the dose-escalation plan statistically significantly boosted the dose delivered to the tumor sub-volumes with high metabolic activities. In the meanwhile, the doses received by OAR including the heart, ipsilateral lung and contralateral lung were not statistically and significantly increased. The results revealed that by using the sub-volume oriented dose-escalation plan, the dose delivered to the target tumor can be boosted while preserving the dose delivered to OAR.

With the proposed method, the accurate segmentation of target tumor volume and measurement of heterogeneous sub-volumes would be beneficial to tumor metabolism analysis, heterogeneous dose calculation and escalation in FDG-PET driven dose painting by contours or numbers so as to improve the local disease control rate (DCR). The proposed model may also contribute to image-centric prediction models such as survival prediction and treatment response prediction following treatment by chemotherapy, radiotherapy, and hypnotherapy. Given the PET images obtained at different scanning time intervals or treatment stages, the proposed technique would be able to quantitatively track the re-distribution of metabolic sub-volumes. For patients with NSCLC, molecularly targeted therapy using epidermal growth factor receptor (EGFR) inhibitors is well developed. Thus, future studies may include FDG PET/CT data before and after targeted therapy and then analyze the dynamic changes of MTV in segmented areas as well as texture parameters. By such, it could continuously monitor and evaluate the therapy responses and thereby could make contributions to personalized treatment plans.

### Limitations

The dose prescriptions in treatment plan B were designed to evaluate the proposed computational technique in dose-escalation to tumor sub-volumes with varying metabolic activities. Although the plan complies with protocol, it is not guaranteed that this is a most optimized plan. As this is a retrospective study, the treatment response using this plan in clinical practices has not been evaluated in this work.

## Conclusion

We proposed an automated target tumor delineation and quantitative tumor heterogeneity analysis approach. The experimental results proved that our method achieved improved segmentation accuracy. The dose-escalation plan using our heterogeneity measurement boosted the tumor sub-volumes with varying metabolic activities while controlling the dose livered to the organs at risk. This technique would offer an efficient way to assist the precise radiation therapy procedures, particularly for the lung cancer patients who need personalized dose painting to achieve high-quality RT results and deal with the local recurrence. The treatment response using the proposed method in clinical practices should be evaluated before its wide application.
